# Nuclear Transporting Factor 2 as a Novel Biomarker of Head and Neck Squamous Cell Carcinoma and Associated with T/B Cell Receptor Signaling Pathway

**DOI:** 10.1155/2022/2885323

**Published:** 2022-02-04

**Authors:** Tingmin Zhang, Yue Xi, Tianfu Wu, Jianfeng Liu

**Affiliations:** The State Key Laboratory Breeding Base of Basic Science of Stomatology (Hubei-MOST) & Key Laboratory of Oral Biomedicine Ministry of Education, School & Hospital of Stomatology, Wuhan University, 430079 Wuhan, China

## Abstract

**Objective:**

This study is aimed at exploring the role of nuclear transporting factor 2 (NUTF2) in head and neck squamous cell carcinoma (HNSCC) based on The Cancer Genome Atlas (TCGA) database.

**Methods:**

We obtained 528 HNSCC patients' clinical data from TCGA and performed expression level analysis of NUTF2. Gene Sets Enrichment Analysis (GSEA) was conducted to identify NUTF2-associated regulatory mechanisms in HNSCC. In addition, several other tools were used to enrich the regulatory network.

**Results:**

We found that NUTF2 was significantly upregulated (*P* < 0.001) in HNSCC. We then observed that higher NUTF2 is associated with poorer overall survival and disease-free survival. Further, by using Cox analyses, we determined high NUTF2 as an independent risk factor of predicting poorer overall survival. Tumor immune infiltration analysis revealed a significantly negative correlation between NUTF2 expression and the level of tumor infiltrated CD8^+^ T cell and B cell, suggesting that NUTF2 may be involved in the immune regulation of HNSCC. Gene sets related to T/B cell receptor signaling pathways were differentially enriched based on the NUTF2 expression phenotype. KEGG pathways were used to show that NUTF2 may affect proliferation, differentiation, and immune response of T/B cell through regulating PI3K/AKT, NF*κ*B, MAPK, and Calcium signaling pathways.

**Conclusion:**

NUTF2 might be a valuable biomarker for HNSCC and correlated with T/B cell receptor signaling pathway.

## 1. Introduction

Head and neck cancers (HNSC) occur in the mucosal linings of the aerodigestive tract, including the lip, oral cavity, salivary glands, pharynx, and larynx. About 900,000 new cases of HNSCC occurred, and over 400,000 patients died from it per year [1, 2]. Head and neck squamous cell carcinomas (HNSCC) account for 90% of HNSC [3]. The prognosis for patients with HNSCC is unfavorable and largely determined by the cancer stage and TNM classification. Typically, the survival rate of patients decreases rapidly with cancer progression [4, 5]. For HPV-positive HNSCC, early viral screening can be used to diagnose high-risk groups. At the same time, the high expression of PD-L1 in HPV-positive HNSCC provides evidence for the feasibility of immunotherapy [6]. Vaccine and drug advances continue to be made, making the prognosis for these patients far better than that for the HPV-negative ones [7]. So far, no evidence-based screening protocols have been validated for all patients with HNSCC [8]. Traditional treatment options for HNSCC including surgical and nonsurgical methods have reached a bottleneck in improving patient survival and prognosis [9]. The advent of immunotherapy is the most remarkable advance in the field of HNSCC treatment in the past decade. For patients with refractory and/or metastatic (R/M) HNSCC, immunotherapy provides an option to maintain long-term remission while other therapies have limited effect [10]. Clinical trials have shown that immunotherapy can be used to enhance the effectiveness of conventional therapies, and this combination therapy will be used in the first line [11]. Limited immunotherapeutic targets were found to be effective, and the immune response to single antigen targeted therapy is at a poor level. Synergy trials are underway, and molecular targeted drugs will be combined with immunologic agents in later stages [10].

NUTF2 (nuclear transporting factor 2, also known as NTF2) is a factor that mediates the import of GDP-bound Ran from the cytoplasm into the nucleus through nuclear pore complexes (NPCs). In previous studies, NUTF2 was the cargo that imported the small GTPase Ran [12]. The process that Ran-GDP bound to NPCs was mediated by NUTF2, which showed that NUTF2 played an essential role in the translocation stage of nuclear protein import [13, 14]. Other results found that the level of NUTF2 could regulate the efficiency of protein transport between nucleus and cytoplasm [15]. In previous studies of nuclear transporters, they were often associated with tumor genesis. In particular, cargo and NPCs play a regulatory role in altered nuclear-cytoplasmic transport patterns in cancer cells [16]. In 2016, Stelma et al. examined the Karyopherin superfamily, a kind of transport receptor, involved in the regulation of cancer through alterations in gene expression [17]. The potential of nuclear transporters as biomarkers and therapeutic targets was highlighted. However, few studies have explored the relationship between NUTF2 and cancers. An experiment in Xenopus laevis cells found that decreased level of NUTF2 may contribute to melanoma development through the regulation of cell nuclear size [18]. On the other hand, in 2020, Du et al. found that NUTF2 is a downstream target of LINC00173 and regulates tumorigenesis in glioma; its overexpression awakens the proliferation, migration, and invasion of cells [19]. The association between NUTF2 and HNSCC has not been illustrated in previous studies.

In this study, we demonstrated NUTF2 expression in HNSCC and investigated its clinical significance. The correlations between NUTF2 expression and tumor infiltrated CD8^+^ T cell and B cell were confirmed. Through using GSEA, we explored the mechanism that may be related to NUTF2 in the regulation of tumor immunity.

## 2. Materials and Methods

### 2.1. Clinical Data from TCGA

528 HNSCC patients' clinical characteristics including age, gender, status, grade, and different cancer classifications were downloaded from TCGA (https://portal.gdc.cancer.gov/). Furthermore, because of the relevance between the NUTF2 expression and clinical message, we obtained the gene expressions of these patients from TCGA. To support further research like CNV analysis, the copy number variation data were also downloaded from TCGA. Before the analysis, incomplete data had been deleted. Detailed information is shown in Table 1.

### 2.2. Statistical Analysis

Statistical analysis was performed by the R software (version 3.6.1 (2019-07-05)). The Wilcoxon rank-sum test was used to compare the expression of NUTF2 in the patient's normal tissues and cancer tissues. In the clinical correlation analyses, the Wilcoxon rank-sum test was used when only two groups of samples were compared, and the Kruskal-Wallis rank-sum test was used when the sample groups were more than two, reaching three or more. Moreover, cox regression analysis was used to determine NUTF2 as a risk factor for HNSCC by using the Survival package of R. Besides, the expression data with |log2 fold change (FC)| was adopted, which made HR more pronounced than raw data. The amendatory *P* values were also used to show the significance of HR. The data of NUTF2 copy number variations (CNVs) in two kinds of tissues was checked by the chi-square test, and the adjusted *P* value was adopted. The Circos 2D track plot was produced by the RCircos package of R [20].

### 2.3. GEPIA

Gene Expression Profiling Interactive Analysis (GEPIA, version 2, http://gepia2.cancer-pku.cn/#index) was a visualized gene expression analytical tool. In this study, NUTF2 expression compared in different subtypes of HNSCC was based on TCGA data from GEPIA, and the boxplot was downloaded from GEPIA. The survival Kaplan-Meier curve was plotted on GEPIA as well.

### 2.4. Gene Set Enrichment Analysis (GSEA)

Gene Set Enrichment Analysis (GSEA, version 3.0, http://software.broadinstitute.org/gsea/index.jsp) is a genomewide expression analytical tool that detects different sets of genes between two biological groups [21]. In this study, GSEA was performed to reveal biological pathways enriched in high-NUTF2 groups. The NUTF2 expression level was used as a phenotype label. The number of gene set permutations was 1,000 for each analysis. Annotated gene sets c2.cp.kegg.v7.0.symbols.gmt were chosen to be the reference gene sets. The nominal *P* value < 0.05 and false discovery rate (FDR) < 0.25 were set as the cut-off criteria.

### 2.5. TIMER

Tumor Immune Estimation Resource (TIMER, version 2.0, http://timer.cistrome.org/) is an enhanced version developed recently based on the original TIMER, which is a web server for comprehensive tumor-immune interactions research [22]. TIMER 2.0 integrates multiple algorithms for immune infiltration estimation and allows users to explore various associations between immune infiltrates and genetic features in the TCGA cohorts [23]. Our results were using the Microenvironment Cell Populations-counter (MCP-counter) method, which allows the robust quantification of the absolute abundance of eight immune and two stromal cell populations in heterogeneous tissues from transcriptomic data [24].

### 2.6. Connectivity Map

Connectivity Map (cMap, version 2.0, also known as L1000, https://clue.io) is an online platform that connects genes, drugs, and disease states by gene expression signatures [25]. This new version of cMap contains 1.3 million L1000 profiles, and its analytical tools allow researchers to discover the mechanism of action of small molecules, functionally annotate genetic variants of disease genes, and inform clinical trials.

## 3. Results

### 3.1. Patient Characteristics in TCGA-HNSCC

As shown in Table 1, the clinical data downloaded from TCGA database consists of 528 HNSCC patients. The median age at diagnosis was about 61 years old. 26.9% patients were male, and 73.1% were female. The living status included 329 (62.3%) dead and 199 (37.7%) alive. Histological grade I could be found in 63 (11.9%) patients, G2 in 311 (58.9%), G3 in 125 (23.7%), G4 in 7 (1.3%), and GX in 18 (3.4%). In tumor classification, 1 (0.2%) in all was T0, 49 (9.3%) were T1, 140 (26.5%) were T2, 101 (19.1%) were T3, 175 (33.1%) were T4, and 39 (7.4%) were TX. In metastasis classification, most patients (*n* = 366, 63.6%) were not sure whether their cancer cells metastasized, 191 (36.2%) were M0, and only 1 (0.2%) was M1. And in regional lymph node classification, node involvement was not observed in 180 (34.1%) patients, 68 (12.9%) were N1, 72 (13.6%) were N2, 8 (1.5%) were N3, and 75 (14.2%) were NX.

### 3.2. Upregulated NUTF2 Expression in HNSCC

**Figure 1:**
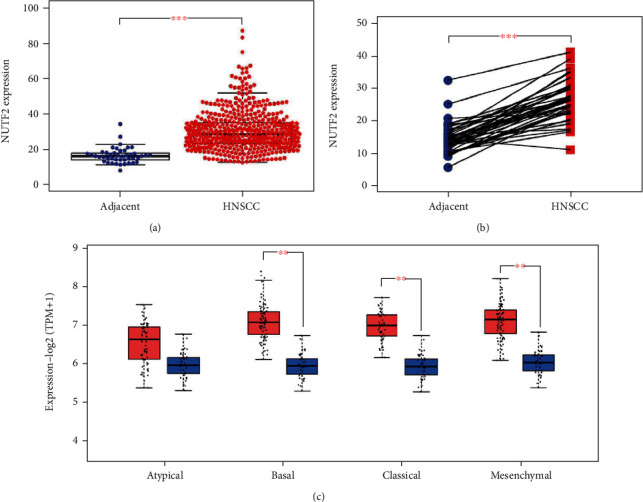
NUTF2 expression in the tumor tissues vs. the adjacent normal tissues. (a) NUTF2 showed significantly higher expression in the tumor tissues than in the adjacent normal tissues; (b) NUTF2 was significantly upregulated in HNSCC compared with the paired noncancerous adjacent tissues from same patient using Wilcoxon rank-sum test; (c) NUTF2 was upregulated significantly in basal, classical, and mesenchymal subtypes of HNSCC. The red box (on the left) represents tumor, and the blue box (on the right) represents normal. Statistical significance compared with the control is indicated by ^∗^*P* < 0.05, ^∗∗^*P* < 0.01, and ^∗∗∗^*P* < 0.001.

**Figure 2:**
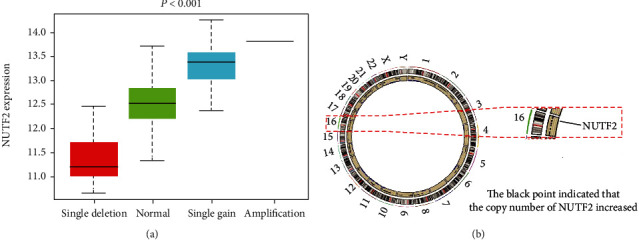
CNV analysis of NUTF2 in HNSCC. (a) Positive correlation between NUTF2 copy number and NUTF2 expression; (b) NUTF2 was located on chromosome 16. The black point at the corresponding site indicated that the copy number of NUTF2 in cancer tissues usually increased.

Compared with the adjacent normal tissues, the results indicated that NUTF2 expression was significantly upregulated in the tumor tissues (Figure 1(a)). Furthermore, the paired analysis, in which normal and tumor tissue come from same patient, showed a significant upregulation of NUTF2 expression in HNSCC (Figure 1(b)). GEPIA database was used to check NUTF2 expression in different subtypes of HNSCC. With four major subtypes of HNSCC, atypical, basal, classical, and mesenchymal, NUTF2 expression in three subtypes was significantly higher in cancer tissues compared with adjacent normal tissues (*P* < 0.01); only the atypical subtype showed no significant difference, but high NUTF2 expression in tumor tissue was still observed in the boxplot (Figure 1(c)). To probe the mechanism of NUTF2 amplification in HNSCC, we examined the copy number variation (CNV) of this gene in patients with HNSCC. A positive correlation between copy number variation and NUTF2 expression was found (Figure 2(a)). Besides, the increased copy number of NUTF2 in HNSCC patients can be observed from the Circos 2D track plot (Figure 2(b)). We concluded that NUTF2 expression was significantly upregulated in HNSCC, which related to the increase of CNV.

### 3.3. Diagnostic and Prognostic Value of NUTF2 Expression in HNSCC

**Figure 3:**
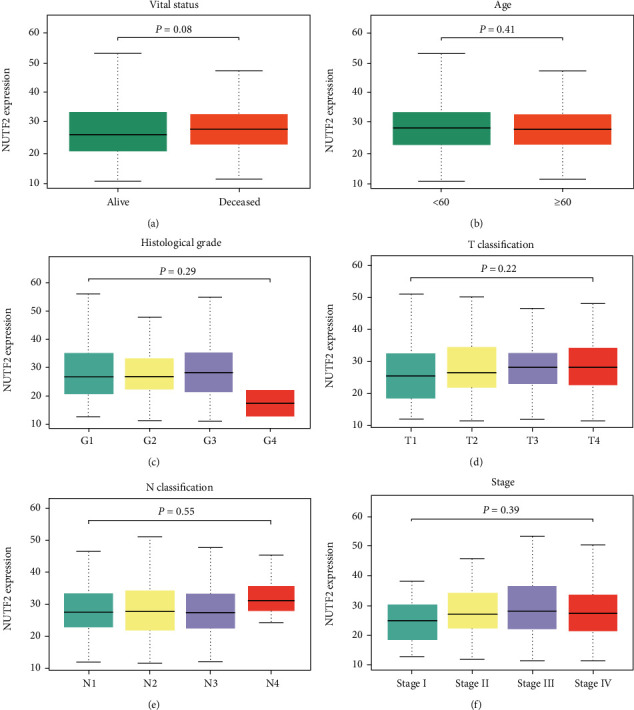
Comparison of NUTF2 expression in HNSCC according to vital status (a), age (b), histological grade (c), T classification (d), N classification (e), and stage (f).

To explore the clinical significance of NUTF2, we compared NUTF2 expression in HNSCC patients according to different clinical features. However, there was no significant difference between different groups in age, TN classification, histological grade, and cancer stage (Figure 3).

**Figure 4:**
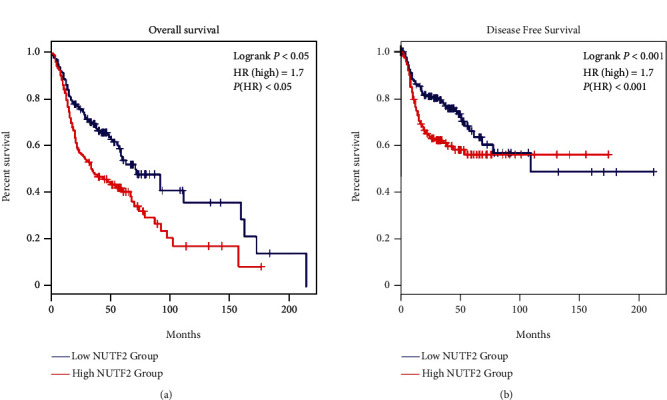
Survival analysis of NUTF2 based on data from TCGA-HNSCC in GEPIA database. The results showed significant correlations between higher NUTF2 expression with poorer overall survival ((a), *P* < 0.05) and Disease Free Survival ((b), *P* < 0.001). HR: hazard ratio.

To assess the prognostic value of NUTF2 for HNSCC, patients were divided into two groups, one with low NUTF2 expression and the other with high NUTF2 expression. We found significant differences between the two groups both in overall survival (OS, Figure 4(a)) and disease-free survival (DFS, Figure 4(b)). The survival rate (both OS (*P* < 0.05) and DFS (*P* < 0.001)) in high NUTF2 group was lower than that in low NUTF2 group. This result indicated that high NUTF2 expression had a positive association with low survival rate of HNSCC patients. Hazard ratio (HR) implied that high NUTF2 expression might have a predictive value for lower survival rates in HNSCC patients (OS: HR = 1.7, *P* < 0.05; DFS: HR = 1.7, *P* < 0.001). To check NUTF2 as a risk factor for HNSCC, the univariate and multivariate cox regression analysis was performed. Through univariate analysis, we confirmed that expression of NUTF2 had a close connection with OS in HNSCC (HR = 1.465, 95% CI = 1.037-2.070, *P* < 0.05). In multivariate analysis with relevant clinical feature adjustment, such as age, gender, grade, stage, T classification, and N classification, the result shown in Table 2 further confirmed that high NUTF2 expression was an independent risk factor associated with significantly worse overall survival (HR = 1.456, 95% CI = 1.020-2.079, *P* < 0.05).

### 3.4. Tumor Immune Infiltration Analysis by TIMER

**Figure 5:**
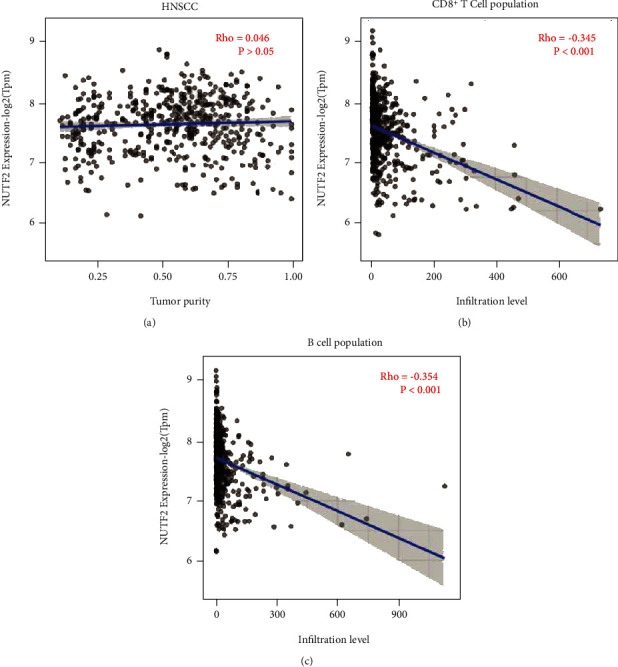
The relationship between NUTF2 expression and immune cell infiltration level. The relationship between tumor purity of HNSCC and NUTF2 expression (a) was shown on left as a control. CD8+ T cell (b) and B cell (c) infiltration levels had negative correlations with NUTF2 expression (Rho < 0, ∣Rho | >0.3, *P* < 0.001).

To uncover the relationship between NUTF2 expression and tumor immune infiltration, Tumor Immune Estimation Resource (TIMER) was used. As the figure showed, the population of tumor infiltrated CD8^+^ T cell and B cell in HNSCC had significantly negative correlations with NUTF2 expression (Figures 5(b) and 5(c), Rho < 0, ∣Rho | >0.3, *P* < 0.05). While the tumor purity of HNSCC showed no relationship with NUTF2 expression, which excluded the interference of NUTF2 on the tumor microenvironment (Figure 5(a)). With tumor purity unregulated, the correlations further illustrated the possible role of NUTF2 in the regulation of tumor infiltrated CD8^+^ T cell and B cell.

### 3.5. Identification of Potential Pathways by GSEA

**Figure 6:**
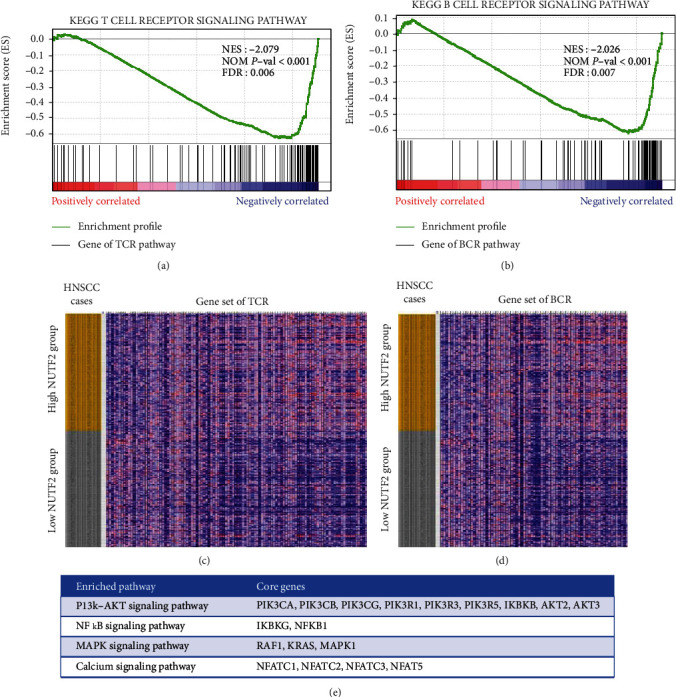
Enrichment plots from GSEA. Gene sets related to T cell receptor signaling pathway (a, c) and B cell receptor signaling pathway (b, d) were differentially enriched in HNSCC cases with low NUTF2 expression (*P* < 0.001). The table illustrated the top 4 pathways enriched among the leading edge subsets. TCR: T cell receptor; BCR: B cell receptor.

**Figure 7:**
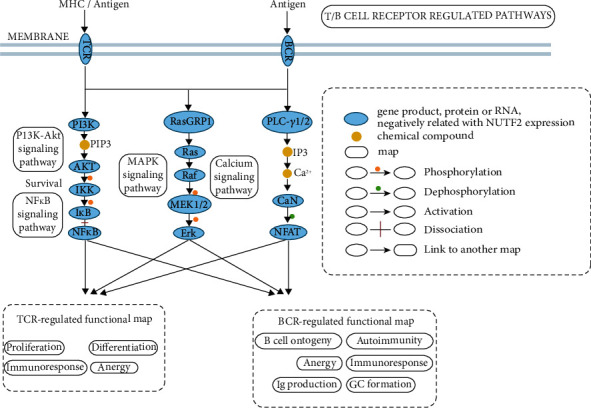
Protein regulation in T/B cell receptor signaling pathways. Core enrichment genes analyzed by GSEA were enriched in four signaling pathways including PI3K/Akt, NF*κ*B, MAPK, and Calcium signaling pathways. TCR and BCR regulate their respective functional modules through these four pathways. TCR: T cell receptor; BCR: B cell receptor.

To identify the altered pathways related to NUTF2 in tumor immunity of HNSCC, Gene Set Enrichment Analysis (GSEA) was conducted between low and high NUTF2 expression groups of HNSCC cases. As Figures 6(a) and 6(b) showed, gene sets related to T cell receptor signaling pathway and B cell receptor signaling pathway were differentially enriched with the low NUTF2 expression phenotype, which suggested NUTF2 negatively regulated T cell receptor signaling pathway and B cell receptor signaling pathway. The relevant genes of T cell receptor signaling pathway and B cell receptor signaling pathway were shown in Figures 6(c) and 6(d). Among them, the core genes of the leading edge subsets which were most closely associated with T/B cell receptor pathway enrichment and the specific pathways they regulate were shown in Figure 6(e). Furthermore, we exhibit the proteins encoded by the enriched core genes in the signaling pathways, and the results demonstrated the negative correlation between these proteins and NUTF2 expression. The proteins encoded by the core enrichment genes were concentrated in PI3K/Akt, NF*κ*B, MAPK, and Calcium signaling pathways, which determined the proliferation, differentiation, anergy, and immune response of T/B cells (Figure 7). This result suggests that the negative role of NUTF2 in the regulation of T/B cells through the above pathways.

### 3.6. Candidate Small Molecule Drugs Based on cMap

To identify potential small molecule drugs targeting NUTF2, Connectivity Map (cMap) was performed to screen out candidate molecule compounds. 9 genes expressed similarly with NUTF2 in HNSCC were detected by GEPIA; then, the 10 upregulated genes were uploaded to Connectivity Map. The output contained the name of compounds, their corresponding connectivity score, target, and mode of action. The connectivity score ranked from -100 to 100. The closer it gets to -100, the more likely it would be the potential drug for HNSCC. The 10 most significant compounds were benzbromarone, mosapride, ITE, azathioprine, orphenadrine, H-9, pergolide, protriptyline, CITCO, and rhamnetin (Table 3).

## 4. Discussion

Previous studies have demonstrated nuclear transporters regulate carcinogenesis by nuclear transporting in cancer cells. However, few studies have explored the topic of NUTF2 with cancer. This study is aimed at demonstrating the role of NUTF2 in HNSCC.

We found a significant upregulation of NUTF2 expression in HNSCC patients, and high NUTF2 expression was associated with poor overall survival. The same result was observed by Huang's research [26]. Besides, we found higher NUTF2 expression in different HNSCC subtypes as well as in paired comparisons of tumor tissue with normal tissue from the same patients. Moreover, we discussed the prognostic value of NUTF2 in HNSCC by DFS analysis, which excludes patients who survive but relapse with HNSCC, offering patients a direct clinical benefit on tumor recurrence and can be a more valuable survival index. Furthermore, univariate and multivariate Cox analyses confirmed NUTF2 expression as an independent risk factor of HNSCC. Based on the results above, NUTF2 is a useful prognostic biomarker of HNSCC.

Early reports showed that NUTF2 engaged in protecting from diabetic retinopathy through depressing vascular endothelial growth factor (VEGF) expression [27]. Moreover, NUTF2 aggregation may alter nucleoplasm transport in nerve cells of Alzheimer's disease patients, leading to neuronal abnormalities and death [28]. RB NLS-mediated nuclear import was critically regulated by NUTF2 [29]. In addition, it was observed that NUTF2 cell localization was different between young cells and senescent cells, suggesting that NUTF2 may influence the cell aging process to some extent [30]. In tumor research, long noncoding RNA (lncRNA) LINC00173 could promote the development of disease by enhancing NUTF2 expression in glioma [19]. Besides, the level of NUTF2 expression was downregulated and negatively correlated with nuclear size in melanoma tissue [18]. NUTF2 overexpression was examined to exhibit melanoma metastasis, cell proliferation, and increase apoptosis. Increasing NUTF2 levels plays a negative role in the progression of melanoma. In addition, NUTF2 was expected to provide a target for combination therapy as a novel treatment for cancers; in prostate cancer cells, it improved drug sensitivity and reduced cell proliferation; in melanoma, it downregulated factors that caused drug resistance [31, 32].

In this study, the tumor immune infiltration analysis suggested negative correlations between NUTF2 expression and infiltrated T cell and B cell levels in HNSCC. Moreover, negative correlations were found between NUTF2 expression and T cell receptor signaling pathway and B cell receptor signaling pathway. As we know, through antigen presentation, T cells and B cells were activated and suppressed the tumor progression [33], and the dysfunction of T cells and B cells leads to the immune escape of tumor cells [34]. Many tumor cells inhibit the effector function of tumor-specific T cells, inducing a state of anergy [35]. T/B cell costimulator is known to activate downstream signaling molecules including NF*κ*B, MAPK, ErK, and nuclear factor of activated T cells (NFAT) [36–38]. The aberrant expression of NUTF2 in T/B cells resulted in the disorder of PI3K/Akt, NF*κ*B, MAPK, and Calcium signaling pathways, which determine the immune response and fate of T/B cells. Our findings suggested that NUTF2 may participate in the immune regulation through regulating T/B cell receptor signaling pathways in the process of tumor oncology in HNSCC.

Besides, cMap analysis was used to search for candidate small molecule drugs. 10 upregulated genes (NUTF2 and other 9 genes detected by GEPIA) in HNSCC were submitted, and the output showed several compounds that have a high negative correlation and potential to treat HNSCC. Among the top 10 compounds, some have been proven to have anticancer effects, such as ITE. ITE is an AhR (aryl hydrocarbon receptor) ligand; the AhR can inhibit cellular proliferation in a ligand-dependent manner and act as a tumor suppressor. In previous studies, ITE was examined to be able to suppress the proliferation and migration of several kinds of cancer cells, such as breast, endogenous, and ovarian cancer cells [39–41]. Moreover, ITE was found to regulate T cell function in inflammation and autoimmune diseases [42, 43]. In addition, pergolide may play an indirect immunomodulatory role by regulating the level of adrenocorticotropic hormone [44]. Therefore, we consider the identified molecule drugs might have the potential to treat HNSCC.

HNSCC is an immunosuppressive type of cancer [45]. Clinical trials have demonstrated that chemotherapy combined with immunotherapy can improve overall survival in patients with HNSCC partly [40]. We found that NUTF2 was associated with the regulation of T/B cell receptor signaling pathways; therefore, it may be a potential target for treatment of HNSCC. In summary, our work showed the role of NUTF2 in HNSCC and explored several possible pathways; however, the specific functions and regulatory mechanism still need further research.

## Figures and Tables

**Table 1:** Patient characteristics in TCGA-HNSCC.

Clinical characteristics	Total 528	%
Age		
61 (average)		
(19-90)		
Gender		
Male	142	26.9
Female	386	73.1
Not available	1	0.2
Status		
Alive	329	62.3
Deceased	199	37.7
Grade		
G1	63	11.9
G2	311	58.9
G3	125	23.7
G4	7	1.3
GX	18	3.4
Not available	4	0.8
Stage		
Stage I	27	5.1
Stage II	74	14.0
Stage III	82	15.5
Stage IV	270	51.1
Not available	75	14.2
T classification		
T0	1	0.2
T1	49	9.3
T2	140	26.5
T3	101	19.1
T4	175	33.1
TX	39	7.4
Not available	23	4.4
M classification		
M0	191	36.2
M1	1	0.2
MX	65	12.3
Not available	271	51.3
N classification		
N0	180	34.1
N1	68	12.9
N2	72	13.6
N3	8	1.5
NX	75	14.2
Not available	25	4.7

**Table 2:** Univariate and multivariate cox analysis of overall survival in patients with HNSCC.

Parameter	Univariate analysis			Multivariate analysis		
	HR	95% CI	*P* value	HR	95% CI	*P* value
Age	1.020	1.004-1.036	0.014	1.025	1.008-1.043	0.003
Gender	0.723	0.506-1.035	0.076	0.798	0.550-1.158	0.235
Grade	1.177	0.903-1.533	0.228	1.026	0.774-1.360	0.857
Stage	1.627	1.279-2.069	≤0.001	1.327	0.887-1.986	0.168
T	1.316	1.104-1.570	0.002	1.028	0.795-1.330	0.838
N	1.527	1.273-1.832	≤0.001	1.375	1.088-1.739	0.008
NUTF2	1.465	1.037-2.070	0.030	1.456	1.020-2.079	0.039

HR: hazard ratio; CI: confidence interval.

**Table 3:** Top 10 most significant compounds based on cMap analysis.

cMap name	Score	Target	MOA
Benzbromarone	-99.93	ABCC1, SLC22A12	Chloride channel blocker
Mosapride	-99.89	HTR4, HTR3A	Serotonin receptor agonist
ITE	-99.89	AHR	Aryl hydrocarbon receptor agonist
Azathioprine	-99.89	HPRT1, IMPDH1, PPAT	Dehydrogenase inhibitor
Orphenadrine	-99.86	CYP2B6, GRIN1, GRIN2D, GRIN3A, GRIN3B, HRH1, SCN10A, SLC6A2	Acetylcholine receptor antagonist
H-9	-99.86	PRKACA	PKA inhibitor
Pergolide	-99.86	DRD1, DRD2, ADRA2A, ADRA2B, ADRA2C, DRD3, DRD4, DRD5, HTR1A, HTR1B, HTR1D, HTR2A, HTR2B, HTR2C, ADRA1A, ADRA1B, ADRA1D, KCNA5	Dopamine receptor agonist
Protriptyline	-99.82	SLC6A2, SLC6A4	Tricyclic antidepressant
CITCO	-99.82	NR1I3	CAR agonist
Rhamnetin	-99.79	ALOX5, MAPK8	HDAC inhibitor

MOA: mode of action.

## Data Availability

The clinical data used to support the findings of this study were obtained from The Cancer Genome Atlas (TCGA) database (https://portal.gdc.cancer.gov/).
